# Hydrophobic *BEA-Type Zeolite Membranes on Tubular Silica Supports for Alcohol/Water Separation by Pervaporation

**DOI:** 10.3390/membranes9070086

**Published:** 2019-07-17

**Authors:** Kyohei Ueno, Saki Yamada, Toshinari Watanabe, Hideyuki Negishi, Takuya Okuno, Hiromasa Tawarayama, Shinji Ishikawa, Manabu Miyamoto, Shigeyuki Uemiya, Yasunori Oumi

**Affiliations:** 1 Department of Oral Biochemistry, Division of Oral Structure, Function and Development, Asahi University School of Dentistry, 1851 Hozumi, Mizuho, Gifu 501-0296, Japan; 2 Faculty of Engineering, Gifu University, 1-1 Yanagido, Gifu, Gifu 501-1193, Japan; 3 Graduate School of Natural Science and Technology, Gifu University, 1-1 Yanagido, Gifu, Gifu 501-1193, Japan; 4 Graduate School of Engineering, Gifu University, 1-1 Yanagido, Gifu, Gifu 501-1193, Japan; 5 Research Institute for Chemical Process Technology, National Institute of Advanced Industrial Science and Technology (AIST), AIST Central 5, 1-1-1 Higashi, Tsukuba, Ibaraki 305-8565, Japan; 6 Frontier Technologies Laboratory, Sumitomo Electric Industries, Ltd., 1, Taya-cho, Sakae-ku, Yokohama, Kanagawa 244-8588, Japan; 7 Organization for Research and Community Development, Gifu University, 1-1 Yanagido, Gifu, Gifu 501-1193, Japan

**Keywords:** *BEA-type zeolite, hydrophobic zeolite membrane, electrophoretic deposition, tubular silica support, secondary growth, hydrothermal synthesis, ethanol, butanol, pervaporation separation, alcohol selectivity

## Abstract

Hydrophobic pure-silica *BEA-type zeolite membranes with large pores were prepared on tubular silica supports by hydrothermal synthesis using a secondary growth method and were applied to the separation of alcohol/water mixtures by pervaporation (PV), an alternative energy-efficient process for production of biofuels. Amorphous pure-silica tubular silica supports, free of Al atoms, were used for preparing the membranes. In this study, the effects of the synthesis conditions, such as the H_2_O/SiO_2_ and NH_4_F/SiO_2_ ratios in the synthetic gel, on the membrane formation process and separation performance were systematically investigated. The successfully prepared dense and continuous membranes exhibited alcohol selectivity and high flux for the separation of ethanol/water and butanol/water mixtures. The pure-silica *BEA membranes obtained under optimal conditions (0.08SiO_2_:0.5TEAOH:0.7NH_4_F:8H_2_O) showed high PV performance with a separation factor of 229 and a flux of 0.62 kg·m^−2^·h^−1^ for a 1 wt % n-butanol/water mixture at 318 K. This result was attributed to the hydrophobicity and large pore size of the pure-silica *BEA membrane. This was the first successful synthesis of hydrophobic large-pore zeolite membranes on tubular supports with alcohol selectivity, and the obtained results could provide new insights into the research on hydrophobic membranes with high permeability.

## 1. Introduction

In the past 20 years, the production of renewable biofuels has received significant attention because of the exhaustion of petroleum resources and the growing interest in environmental issues. Bio-alcohols (bioethanol and biobutanol) produced by fermentation of biomass are examples of biofuels, and are expected to be used as petroleum substitute fuels. However, as the bio-alcohols are essentially dilute solutions (the concentration of bioethanol is 10 wt % or less, and that of biobutanol is 0.5–1.5 wt %), it is necessary to concentrate and purify them for industrial use. Pervaporation (PV) separation is a suitable alternative to the conventional and energy-intensive distillation processes. For recovery of alcohol from the dilute solution in the PV process, a hydrophobic membrane is preferred from the energy point of view because it can preferentially permeate organic matter over water.

As membranes made from inorganic microporous materials, zeolite membranes have attracted considerable attention owing to their good separation performance and higher thermal, mechanical, and chemical stability than that of polymeric membranes [1,2]. Zeolite is a crystalline aluminosilicate, which has unique molecular-sized pores, and its adsorption characteristics (hydrophilic/hydrophobic) can be controlled by the ratio of Si and Al constituting its framework. In particular, membranes made from silicalite-1, which is a pure-silica MFI-type zeolite, exhibit high hydrophobicity, and thus are widely studied as hydrophobic separation membranes for organic matter recovery from dilute solutions, especially ethanol/water mixtures [3,4,5,6,7,8,9,10,11,12,13,14,15,16,17]. However, for industrial applications, it is necessary to prepare a membrane with sufficient selectivity and high permeability. Although silicalite-1 membranes show high separation selectivity for ethanol and butanol separation, their low permeability is one of the problems when aiming at industrialization. One of the reasons for the low permeability of these membranes is the pore size of the MFI-type zeolite (approximately 0.55 nm), which is close to the molecular size of alcohol (ethanol: 0.43 nm; n-butanol: 0.50 nm); thus, the permeation resistance is large when these molecules pass through the zeolite pores. In particular, in the case of butanol/water separation, the permeation flux is very low [12,13,14]. The reason is that the pore size of silicalite-1 and the molecular size of butanol are very similar. Therefore, the utilization of a pure-silica zeolite having larger pores and exhibiting hydrophobicity as a membrane material may contribute to the improvement in the permeation performance of these separation systems.

The *BEA-type zeolite is one of the most important zeolites because of its excellent properties, high thermal stability, and unique large pore structure, which can be described as a three-dimensionally intersected channel system with straight 12-membered ring pores (0.66 nm × 0.67 nm) along the a and b axes and a tortuous channel (0.56 nm × 0.56 nm) along the c axis [18,19]. *BEA zeolite is one of the zeolites whose hydrophilicity and hydrophobicity can be controlled by changing the amount of Al present in the structure. Several *BEA membranes for separation have been developed and they are reported to exhibit excellent membrane performance [20,21,22]. However, most of these membranes contain a large amount of Al in the framework. The hydrophobic pure-silica *BEA zeolite has a very narrow synthesis range and its synthesis and membrane formation are difficult [23,24,25,26]. Thus, the only reported example of a pure-silica *BEA film and membrane is on a flat-type support, which is easy to synthesize [26,27,28]. To the best of our knowledge, no studies applying pure-silica *BEA membranes on tubular supports, which have larger surface areas and are thus better suited for industrial production for alcohol/water separation, have been reported.

Therefore, the purpose of this study is to investigate the synthesis conditions of a pure-silica *BEA membrane on a tubular silica support and the applicability of the prepared *BEA membranes to alcohol/water separation. For preparation of pure-silica *BEA membranes, a novel tubular silica support composed of pure silica was used to prevent the incorporation of Al from the support. The use of a silica support can be expected to produce a high-quality pure silica *BEA membrane because there is no influence of thermal strain between the support and the zeolite membrane layer, and there is no change in the membrane characteristics due to Al component elution from the support [15,16,17]. In this study, pure-silica *BEA membranes were prepared using the hydrothermal synthesis method, and the effects of the synthesis conditions, such as the H_2_O/SiO_2_ and NH_4_F/SiO_2_ ratios in the synthetic gel, on the membrane formation process were investigated. Furthermore, PV tests of ethanol/water and butanol/water mixtures were performed using the obtained membranes to evaluate their applicability.

## 2. Materials and Methods

### 2.1. Classification of *BEA-Type Zeolite Crystals

Pure silica *BEA-type zeolite seed crystals HSZ-990 HOA (Tosoh Corp., Tokyo, Japan) were used. Before using the *BEA-type zeolite as seed crystals, water classification was performed to remove aggregates and obtain uniform crystals. First, 0.2 g of crystals and 40 mL of water were put in a centrifuge tube and centrifuged at 800 rpm for 5 min. After that, the supernatant liquid was transferred to another centrifuge tube and centrifuged at 9000 rpm for 9 min. The finally obtained crystals were used as the seed crystals.

### 2.2. Preparation of Seeded Support by Electrophoretic Deposition (EPD)

The *BEA seed crystals obtained by classification were coated onto a tubular silica support (length: 40 mm; outer diameter: 8.6 mm; inner diameter: 6 mm; porosity: 64%; pore size: 0.5 μm; Sumitomo Electric Industries, Osaka, Japan) using EPD as a seeding method [10]. Methanol and toluene were used as dispersion solvents (methanol/toluene = 95/5 vol %; FUJIFILM Wako Pure Chemical Corp., Osaka, Japan). The EPD bath was prepared by adding the seed crystals to the solvent to obtain a concentration of 5 g·L^−1^ followed by dispersion via ultrasonic vibration for 30 min. In the EPD process, direct current voltage of 100 V was applied for 3 min. The prepared seeded supports were calcined at 573 K for 6 h to enhance adhesion between the silica support surface and the *BEA seed crystals.

### 2.3. Preparation of Pure-Silica *BEA-Type Zeolite Membranes

The synthetic gel composition with SiO_2_:TEAOH:NH_4_F:H_2_O molar ratio of 0.08–1:0.5:0.45–0.7:8 is that presented in a previous report [23]. The synthetic gel was prepared with fumed silica (Cab-O-Sil M-5, Cabot Corp., Boston, MA, USA) and *BEA seed crystals (HSZ-990 HOA, rate of 3 wt % to the silica source) as a silica source, tetraethylammonium hydroxide (TEAOH, 35 wt % in water; SACHEM Japan, Osaka, Japan) as an alkali source, structure-directing agent (SDA) ammonium fluoride (NH_4_F, 97 wt %; FUJIFILM Wako Pure Chemical Corp., Osaka, Japan) as a fluoride source, and distilled water. After stirring at room temperature for 2 h, the hydrogel and the seeded support were introduced into a Teflon-lined stainless-steel autoclave, in which the tubular seeded support was placed vertically. The static hydrothermal synthesis was carried out at 443 K for 24 h for secondary growth of the seed layer. After the reaction, the membrane was washed with distilled water, dried at 333 K overnight, and then calcined at 753 K for 12 h to remove the SDA from the zeolite pores.

### 2.4. Characterization

The obtained samples after calcination were analyzed by X-ray diffraction (XRD; D8 Advance, Bruker AXS, Karlsruhe, Germany), and their morphologies were observed by scanning electron microscopy (SEM; S-4800, Hitachi High-Technologies, Tokyo, Japan). The performance of the obtained membranes was evaluated by PV separation for ethanol/water mixtures (10/90 wt %) at 323 K and n-butanol/water mixtures (1/99 wt %) at 318 K using the system shown in Figure S1. The permeate vapor was collected using a liquid nitrogen-filled cold trap. The alcohol concentrations of the feed and permeate were determined by liquid chromatography (Shimadzu, Tokyo, Japan). The total flux (*J*) is defined as

*J* = *m*/(*A*·*t*) (1)

where *m* is total amount of permeation (kg), *A* is the effective membrane area (m^2^), and *t* is the operating time (h). The separation factor (α) is defined as

α = (*Y*_alcohol_/*Y*_water_)/(*X*_alcohol_/*X*_water_) (2)

where *X* and *Y* are the weight fractions of the species in the feed and permeate, respectively.

## 3. Result and Discussion

### 3.1. Seeded Support

**Figure 1:**
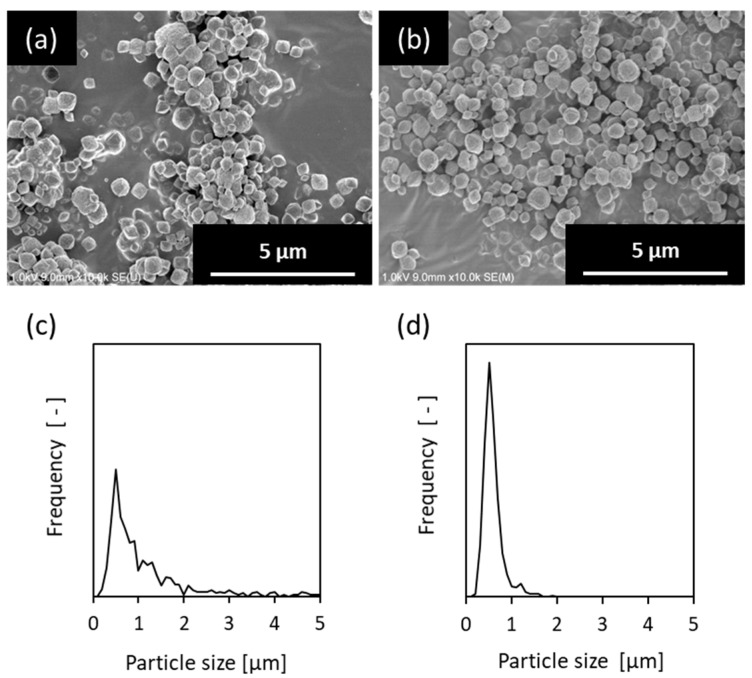
SEM images of seed crystals and particle size distributions derived from the SEM images (**a**,**c**) before and (**b**,**d**) after classification.

The formation of a uniform and continuous seed layer on the support is an important factor in the formation of dense and continuous zeolite membranes [9,10,11,29,30]. Therefore, it is preferable to use a uniform particle diameter of the seed crystal. Figure 1 shows SEM images of seed crystals before and after classification and particle size distributions derived from the SEM images. Before classification, aggregates of seed crystals were observed and showed a broad distribution (Figure 1a,c). After classification, large aggregate crystals were removed, and uniform crystals with an average particle size of approximately 700 nm were obtained (Figure 1b,d). A seeded support was prepared using this seed crystal.

**Figure 2:**
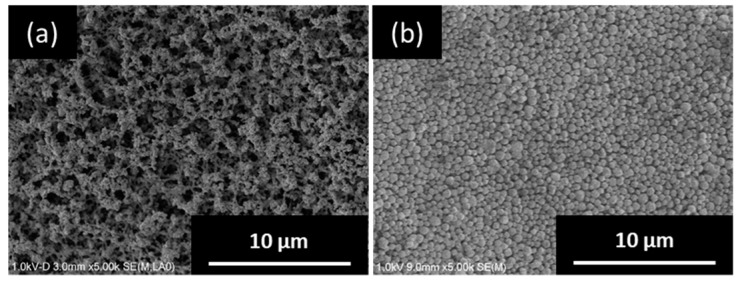
SEM images of the top surface of silica support (**a**) before and (**b**) after seeding.

Figure 2 shows SEM images of the top surface of the silica support before and after seeding by EPD. It can be seen that the silica support has an open-pore structure, which is formed by the sintering of the submicron-sized amorphous silica particles (Figure 2a). After seeding by EPD, the support surface was completely covered with seed crystals and a uniform and continuous seed layer was obtained (Figure 2b). The subsequent membrane synthesis was performed using these seeded supports.

### 3.2. Pure-Silica *BEA-Type Zeolite Membrane

#### 3.2.1. Effect of H_2_O/SiO_2_ Ratio of Synthesis Gel

Generally, the precursor gel used for pure-silica *BEA powder synthesis has a low H_2_O/SiO_2_ ratio and high viscosity [24,25]. However, a precursor gel with high viscosity tends to be nonuniform and leads to a reduction in reproducibility, so it is not preferable to use it as a precursor gel for membrane synthesis. Thus, to increase the H_2_O/SiO_2_ ratio, the amount of SiO_2_ in the precursor gel was reduced (Table 1).

**Figure 3:**
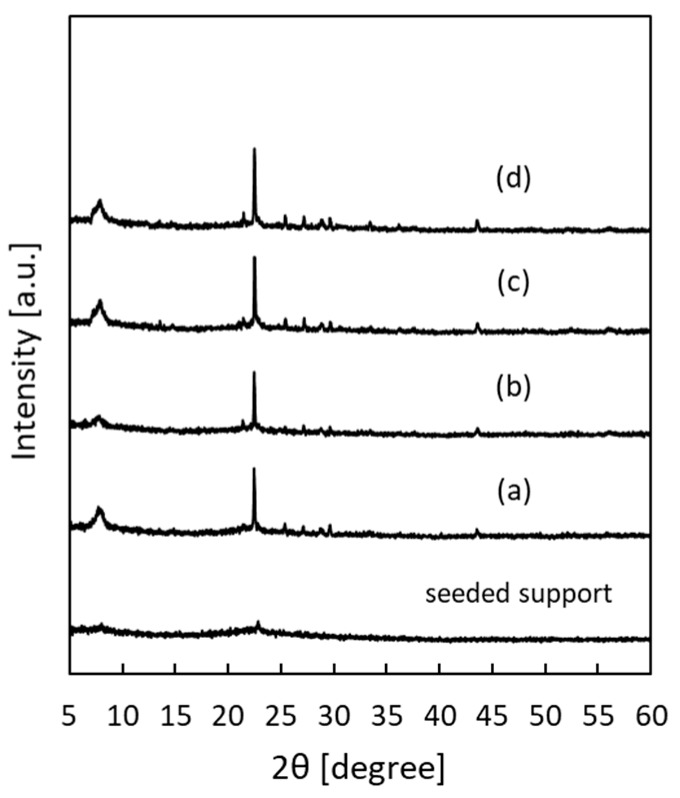
X-ray diffraction (XRD) patterns of *BEA-type membranes prepared with different H_2_O/SiO_2_ ratios: (**a**) 8, (**b**) 40, (**c**) 100, and (**d**) 160.

**Figure 4:**
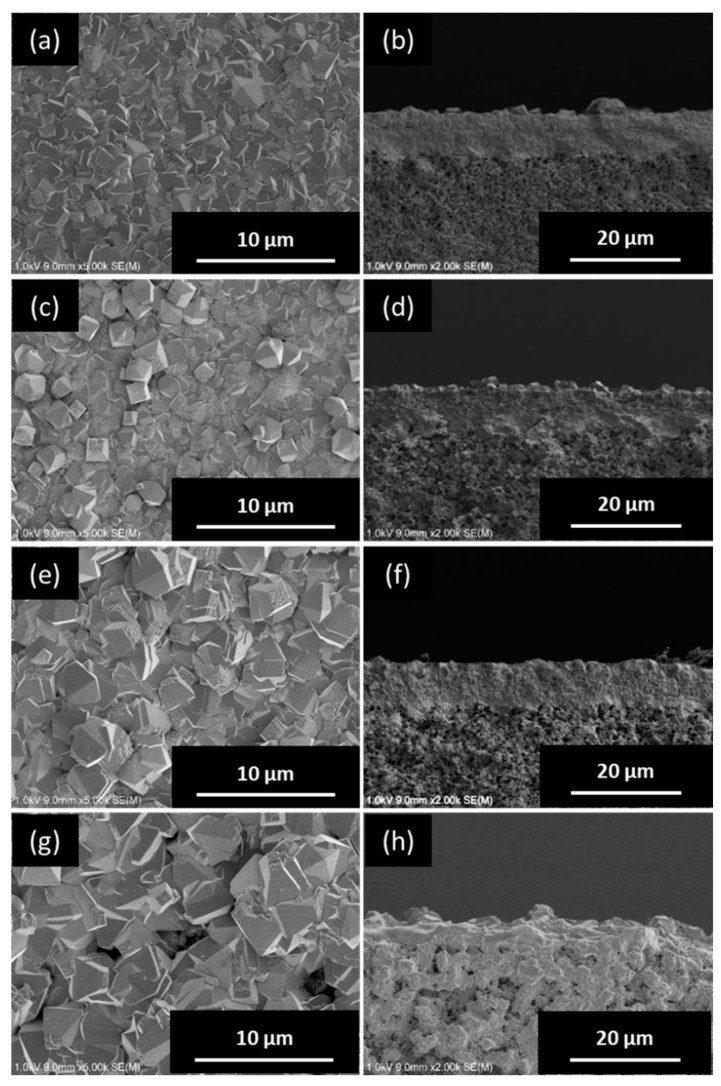
Top (left) and cross-sectional (right) SEM images of *BEA-type membranes prepared with different H_2_O/SiO_2_ ratios: (**a**,**b**) 8, (**c**,**d**) 40, (**e**,**f**) 100, and (**g**,**h**) 160.

Figure 3 shows the XRD patterns of the *BEA membranes prepared with different H_2_O/SiO_2_ ratios. The peak intensity was greatly increased in all samples as compared to the seeded support. In addition, only a peak specific to *BEA-type zeolite was confirmed, and the formation of other impurity layers was not confirmed. The synthesis conditions for pure-silica *BEA-type zeolite crystals are very narrow and, until now, they have been synthesized only from precursors with low water content [24,25]. However, it was found that when a secondary growth was used in this study, it was possible to grow *BEA crystals in a wide range of H_2_O/SiO_2_ ratios. Figure 4 shows the SEM images of the *BEA membranes prepared with different H_2_O/SiO_2_ ratios. From the SEM image (Figure 4), the formation of a zeolite layer having a similar thickness on the support was confirmed with H_2_O/SiO_2_ ratios in the range of 8 to 100. These results indicate that it is possible to form a pure-silica *BEA membrane on a silica support over a wide range of H_2_O/SiO_2_ ratios. However, when the gel with H_2_O/SiO_2_ ratio = 8 (highest viscosity among the gels used) was used, *BEA membrane was not obtained with high reproducibility. In addition, the white suspension in the autoclave after hydrothermal synthesis of *BEA membranes was thicker in color than before hydrothermal treatment and that obtained under other conditions, indicating that the frequency of nucleation in the hydrogel under hydrothermal synthesis was higher. That is, when a high viscosity synthesis gel is used, crystal formation in the hydrogel occurs predominantly rather than the growth of the seed crystal layer on the support, resulting in the membrane thickness failing to increase. Therefore, the reproducibility of the membrane synthesis is low. However, when the H_2_O/SiO_2_ ratio was increased to 160, the membrane layer was not uniform and continuous, and the surface of the support was exposed. This seems to be because the intergrowth between seed crystals could not grow sufficiently because the amount of silica source in synthetic gel was diluted.

The separation performance of the obtained pure-silica *BEA membrane was evaluated by a PV test using an ethanol/water mixture (Table 1). The membranes having the same membrane thickness synthesized at a H_2_O/SiO_2_ ratio = 8–100 showed ethanol selectivity and a similar separation factor. This result demonstrated for the first time that pure-silica *BEA membranes exhibit hydrophobic properties and have ethanol selectivity in the PV test. Table 2 summarizes the ethanol/water separation performances of the silicalite-1 membranes on tubular supports reported in the literature. These membranes prepared in this study have significantly improved permeability but lower selectivity compared to conventional silicalite-1 membranes. Further, the membrane prepared with H_2_O/SiO_2_ = 160 showed almost no separation selectivity owing to the discontinuity of the membrane layer. From the above results, it was found that a hydrophobic *BEA membrane could be synthesized by a dilute gel (H_2_O/SiO_2_ = 100) as compared to the synthesis conditions of the conventional pure-silica *BEA zeolite [23,24].

#### 3.2.2. Effect of NH_4_F/SiO_2_ Ratio of Synthesis Gel

Generally, in the synthesis of pure-silica *BEA zeolite powders, the use of fluoride is essential and contributes to the suppression of defects and the improvement in crystallinity [31]. However, the influence of fluoride on the formation of a pure silica *BEA membrane has not been reported yet. Therefore, the H_2_O/SiO_2_ ratio was fixed at 100, and the influence on the membrane formation process was examined by changing the amount of NH_4_F (Table 3).

**Figure 5:**
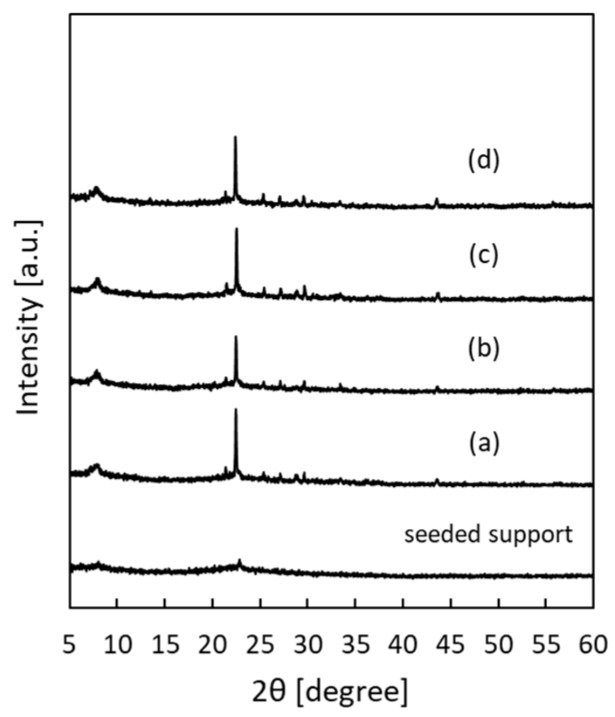
XRD patterns of *BEA-type membranes prepared with different NH_4_F/SiO_2_ ratios: (**a**) 5.63, (**b**) 6.25, (**c**) 7.50, and (**d**) 8.75.

**Figure 6:**
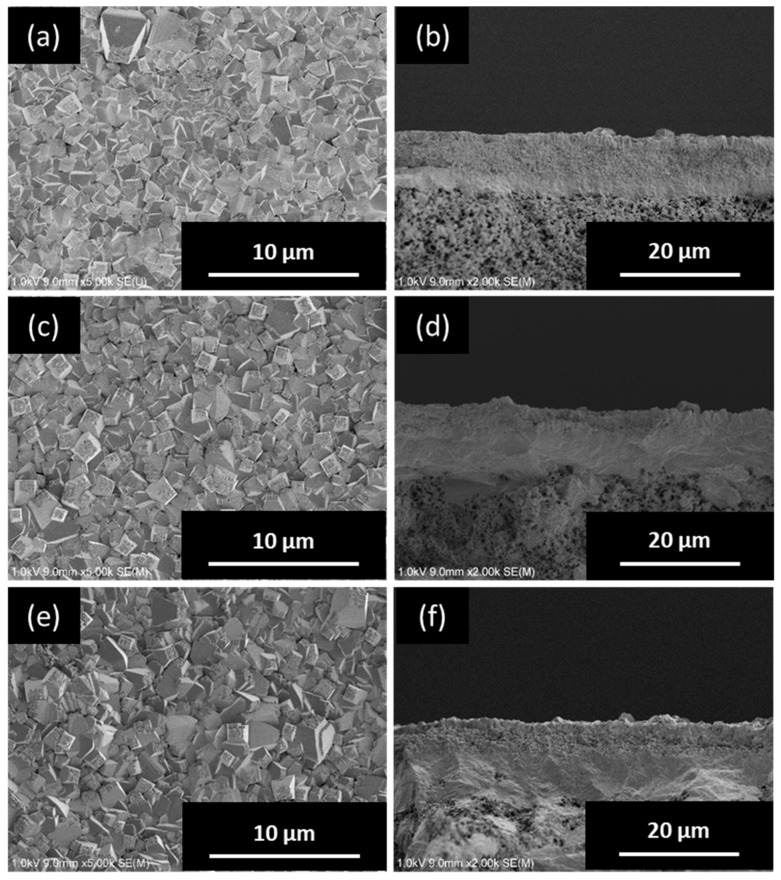
Top (left) and cross-sectional (right) SEM images of *BEA-type membranes prepared with different NH_4_F/SiO_2_ ratios: (**a**,**b**) 6.25, (**c**,**d**) 7.50, and (**e**,**f**) 8.75.

Figure 5 shows the XRD patterns of the *BEA membranes prepared with different NH_4_F/SiO_2_ ratios. The peak intensity was greatly increased in all samples as compared to the seeded support. In addition, only a peak specific to *BEA-type zeolite was confirmed, and the formation of other impurity layers was not confirmed. From the cross-section of the SEM images (Figure 6), the membrane thickness increased with the increase in the NH_4_F/SiO_2_ ratio. Interestingly, the formation of a dense zeolite layer was observed at the interface between the SiO_2_ support and membrane layer when the NH_4_F/SiO_2_ ratio was increased above 6.25. Furthermore, its thickness increased with the increase in the NH_4_F/SiO_2_ ratio. The formation of this dense zeolite layer has also been observed in the synthesis of silicalite-1 membranes on the same SiO_2_ supports and is reported to function as the main separation layer in the separation of ethanol/water mixtures [15]. This dense layer is considered to be formed by the dissolution of the silica support during the hydrothermal treatment, and the result is the increase in the silica concentration in the vicinity of the support. Thus, the use of a silica support creates a silica-rich region (low H_2_O/SiO_2_) near the support. Therefore, it was suggested that an area where the *BEA zeolite is easily synthesized is thus created, and the formation of a dense *BEA membrane layer is possible. In this experiment, it is considered that NH_4_F acts as a mineralizer and increases the thickness of the dense zeolite layer because it promotes crystal growth in the vicinity of the support. Thus, dissolution of the silica support also seems to work effectively in membrane synthesis, even in *BEA membrane synthesis.

The separation performance of the obtained pure-silica *BEA membrane was evaluated by a PV test using an ethanol/water mixture (Table 3). The separation factor improved with the increase in the NH_4_F/SiO_2_ ratio from 5.63 to 7.50. This result is also consistent with the results for silicalite-1 membranes where the separation factor for ethanol/water separation increases with increasing thickness of the dense zeolite layer [15]. However, when the NH_4_F/SiO_2_ ratio was further increased, the separation performance was constant. It is considered that the thickness of the membrane layer is not a direct factor governing the separation performance in ethanol/water separation when using the *BEA membrane.

#### 3.2.3. Separation Performance of Butanol/Water Mixtures

The PV test was conducted using n-butanol/water mixtures. Biobutanol is also a good renewable and clean bio-fuel. The concentration of biobutanol from the fermentation broth is important because of the higher energy density and lower volatility of biobutanol compared to that of bioethanol [14]. For 1 wt % butanol solution at 318 K, the separation factor of butanol improved with the increase of the NH_4_F/SiO_2_ ratio (Table S1), and the membrane named M7 exhibited highest butanol selectivity and showed a separation factor of 229 and a flux of 0.62 kg·m^−2^·h^−1^. Compared to ethanol/water separation, in butanol/water separation the separation factor increased and the flux decreased. This result is also in agreement with previous reports [13,14]: the butanol molecule is more hydrophobic and of larger diameter than that of ethanol. Table 4 summarizes the membranes obtained in this study and the results of butanol/water separation of some high-performance membranes previously reported in the literature. Polymeric membranes and mixed-matrix membranes (MMM) exhibit relatively high permeability to butanol separa0tion, but low selectivity [32,33,34,35]. The silicalite-1 membrane showed relatively high selectivity, but low permeability [12,13,14]. It is considered that the low permeability of silicalite-1 membranes is caused by the similar size of butanol and the silicalite-1 pores. In terms of both the separation factor and permeation flux, the *BEA membrane exhibited excellent PV performance for butanol/water separation. It is believed that this result is due to the high hydrophobicity and large pore size of pure-silica *BEA membranes, which promote the applicability of *BEA membranes for butanol/water separation.

The pure-silica *BEA membrane with large pores obtained in this study has high stability compared to the conventional Al-containing *BEA membrane. Moreover, potential applications in other separation systems, such as separation of large hydrocarbons, can also be expected. Furthermore, although the synthesis time of the conventional pure-silica *BEA membrane required 9 days [27], in this study, we succeeded in synthesizing the pure-silica *BEA membrane in only 1 day. In consideration of the effectiveness of this membrane, further optimization of the synthesis conditions, such as synthesis time and temperature, is necessary to improve the performance by formation of dense and thin membranes.

### 3.3. Separation Mechanism of Hydrophobic *BEA Membrane

**Figure 7:**
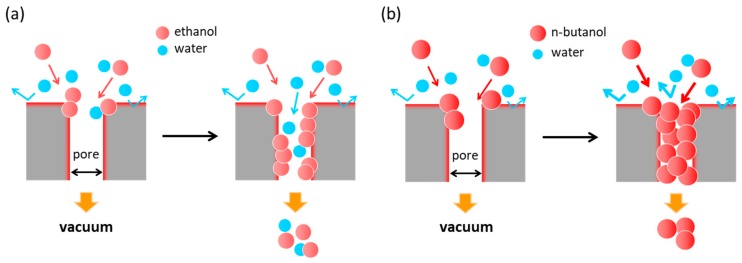
Schematic of separation mechanism of hydrophobic *BEA membrane: (**a**) ethanol/water separation (ethanol molecule << *BEA pore) and (**b**) butanol/water separation (butanol molecule < *BEA pore).

The hydrophobic *BEA membrane proved to be selective to ethanol and butanol in the separation in each case of the aqueous solution by PV and to have a high flux. However, for ethanol/water separation, the separation factor of ethanol was lower than that previously reported for silicalite-1 membranes [3,4,5,6,7,8,9,10,11,12,13,14,15,16,17]. In general, in the case of ethanol/water separation using silicalite-1 membranes, ethanol molecules (0.43 nm) are preferentially adsorbed on the MFI-type zeolite pores (approximately 0.55 nm), thus blocking the zeolite channels. Therefore, the permeation of water molecules is hindered, ethanol molecules are more selectively permeated, and a high separation factor is obtained. However, when a large pore zeolite membrane such as the *BEA-type zeolite (approximately 0.66 nm) is used for ethanol/water separation, the difference between the ethanol molecular diameter and the *BEA pore is large and the pores cannot be filled with the adsorbed ethanol molecules (Figure 7a). Therefore, the permeation of water cannot be prevented, and the separation factor of ethanol has a low value. Similar results are reported in the literature [38], and when using a super-hydrophobic membrane (HiPAS membrane) with large pores (5–100 nm), a high permeation flux can be obtained but the separation factor is low. Thus, it has been found that even if a hydrophobic membrane is prepared, it is difficult to recover ethanol preferentially when there is a large difference between the size of the permeable molecule and the membrane pore. However, in butanol/water separation, butanol molecules have higher adsorption power and larger molecular diameter (0.50 nm) than ethanol. Thus, it is considered that the adsorbed butanol molecules can block the large *BEA channel (Figure 7b). Therefore, the permeation of water molecules is hindered and a high separation factor is obtained. Moreover, because of the large pore size of the *BEA membrane, it shows a much higher flux than that of the silicalite-1 membrane. Thus, it was found that the *BEA membrane has potential as a separation membrane for selectively recovering butanol.

From these results, it was revealed that the size of the permeating molecule and the size of the zeolite pore significantly affect the separation performance, even in PV separation using the difference in adsorptivity by the alcohol-selective hydrophobic membrane. Therefore, it is suggested that the selection of a zeolite having an appropriate pore size is important in synthesizing a high-performance membrane, depending on the molecular diameter of the molecule to be separated.

## 4. Conclusions

Hydrophobic pure-silica *BEA-type zeolite membranes with large pores were prepared on a tubular silica support by hydrothermal synthesis using a secondary growth method and were applied to separation of alcohol/water mixtures by PV for the first time. Amorphous tubular silica supports, which were Al-free, were used for the preparation of the pure-silica *BEA membrane. In this study, the effects of the synthesis conditions, such as the H_2_O/SiO_2_ and NH_4_F/SiO_2_ ratios in the synthetic gel, on the membrane formation process and separation performance were systematically investigated. Dense and continuous pure-silica *BEA membranes were successfully prepared on silica supports, and exhibited alcohol selectivity and high flux for the separation of ethanol/water and butanol/water mixtures. In addition, even in PV separation using an alcohol-selective membrane that utilizes the difference in adsorptivity, it was revealed that the size of the permeation molecules and the size of the zeolite pore significantly affect the separation performance. That is, when the *BEA membrane was used, the selectivity of ethanol was low but the selectivity of butanol was high. Compared to those in the literature, the *BEA membrane obtained under optimal conditions (0.08SiO_2_:0.5TEAOH:0.7NH_4_F:8H_2_O) showed a high PV performance with separation factor of 229 and a flux of 0.62 kg·m^−2^·h^−1^ for a 1 wt % butanol/water mixture at 318 K. This result was attributed to the high hydrophobicity and large pore size of the pure-silica *BEA zeolite membrane. This is the first successful synthesis of hydrophobic large-pore zeolite membranes on a tubular support exhibiting alcohol selectivity, and it is considered that the results provide new insights for the research on hydrophobic membranes with high permeability. In addition, studies on the development of a novel synthetic procedure to reduce the cost of membrane production for industrial applications are underway.

## Figures and Tables

**Table 1:** Effect of H_2_O/SiO_2_ ratio of synthesis gel on the PV performance for ethanol/water mixtures.

Samples	Synthesis Gel Composition ^1^		Separation Factor	Flux [kg·m^−2^·h^−1^]
	x	H_2_O/SiO_2_ Ratio		
M1	1.00	8	8.7	8.56
M2	0.20	40	8.7	7.51
M3	0.08	100	8.5	7.97
M4	0.05	160	1.5	20.6

^1^ SiO_2_:TEAOH:NH_4_F:H_2_O = x:0.5:0.45:8.0.

**Table 2:** Summary of PV performances of silicalite-1 membranes on tubular support for ethanol/water mixtures.

Support Materials	Feed Concentration [wt %]	Feed Temperature [K]	Separation Factor	Flux [kg·m^−2^·h^−1^]	Ref.
α-Al_2_O_3_	3	353	33	0.35	[5]
Silica	3	333	68	0.87	[5]
α-Al_2_O_3_	5	333	85	1.36	[6]
Mullite	5	333	66	1.91	[7]
α-Al_2_O_3_	5	323	76	1.05	[8]
α-Al_2_O_3_	10	323	88	0.47	[10]
α-Al_2_O_3_	5	333	39	1.51	[13]
Silica	10	323	92	3.00	[15]

**Table 3:** Effect of NH_4_F/SiO_2_ ratio of synthesis gel on the PV performance for ethanol/water mixtures.

Samples	Synthesis Gel Composition ^2^		Separation Factor	Flux [kg·m^−2^·h^−1^]
	y	NH_4_F/SiO_2_ Ratio		
M3	0.45	5.63	8.5	7.97
M5	0.50	6.25	9.8	7.37
M6	0.60	7.50	12.3	6.29
M7	0.70	8.75	12.0	6.22

^2^ SiO_2_:TEAOH:NH_4_F:H_2_O = 0.08:0.5:y:8.0.

**Table 4:** Summary of PV performance of various types of membrane for butanol/water mixtures.

Membrane Type	Feed Concentration [wt %]	Feed Temperature [K]	Separation Factor	Flux [kg·m^−2^·h^−1^]	Ref.
PDMS	1.5	328	43	0.67	[32]
PDMS	1	333	51	1.08	[33]
ZIF-7/PDMS	1	333	66	1.69	[33]
ZIF-8/PDMS	1.5	353	82	4.85	[34]
ZIF-8/PMPS	1	353	40	6.40	[35]
Silicalite-1/PDMS	1	313	92	0.13	[36]
Ge-ZSM-5	5	303	19	0.02	[37]
Silicalite-1	1	343	150	0.10	[13]
Silicalite-1	1	318	465	0.04	[12]
Silicalite-1	1.5	353	207	0.22	[14]
*BEA	1	318	229	0.62	This work

## Supplementary Materials

The following are available online at https://www.mdpi.com/2077-0375/9/7/86/s1. Figure S1: Experimental instrument used for measuring pervaporation performance, Table S1: Effect of NH_4_F/SiO_2_ ratio of synthesis gel on the PV performance for butanol/water mixtures.
