# Supplementary material for: Hydrophobic *BEA-Type Zeolite Membranes on Tubular Silica Supports for Alcohol/Water Separation by Pervaporation
Source: Membranes (Basel). 2019 Jul 17;9(7):86. doi: 10.3390/membranes9070086 (PMC6680943; doi:10.3390/membranes9070086)
Supplement: Supplementary file 1 [file membranes-09-00086-s001.pdf]

Supplementary Material

Hydrophobic *BEA-Type Zeolite Membranes on Tubular Silica Supports for Alcohol/Water Separation by Pervaporation

Kyohei Ueno [1, 2], Saki Yamada [3], Toshinari Watanabe [4], Hideyuki Negishi [5], Takuya Okuno [6], Hiromasa Tawarayama [6], Shinji Ishikawa [6], Manabu Miyamoto [2], Shigeyuki Uemiya [2], and Yasunori Oumi [7,*]

[1] Department of Oral Biochemistry, Division of Oral Structure, Function and Development, Asahi University School of Dentistry, 1851 Hozumi, Mizuho, Gifu 501-0296, Japan
[2] Faculty of Engineering, Gifu University, 1-1 Yanagido, Gifu, Gifu 501-1193, Japan
[3] Graduate School of Natural Science and Technology, Gifu University, 1-1 Yanagido, Gifu, Gifu 501-1193, Japan
[4] Graduate School of Engineering, Gifu University, 1-1 Yanagido, Gifu, Gifu 501-1193, Japan
[5] Research Institute for Chemical Process Technology, National Institute of Advanced Industrial Science and Technology (AIST), AIST Central 5, 1-1-1 Higashi, Tsukuba, Ibaraki 305-8565, Japan
[6] Frontier Technologies Laboratory, Sumitomo Electric Industries, Ltd., 1, Taya-cho, Sakae-ku, Yokohama, Kanagawa 244-8588, Japan
[7] Organization for Research and Community Development, Gifu University, 1-1 Yanagido, Gifu, Gifu 501-1193, Japan
* Correspondence: oumi@gifu-u.ac.jp; Tel.: +81-58-293-3335

Received: date; Accepted: date; Published: date

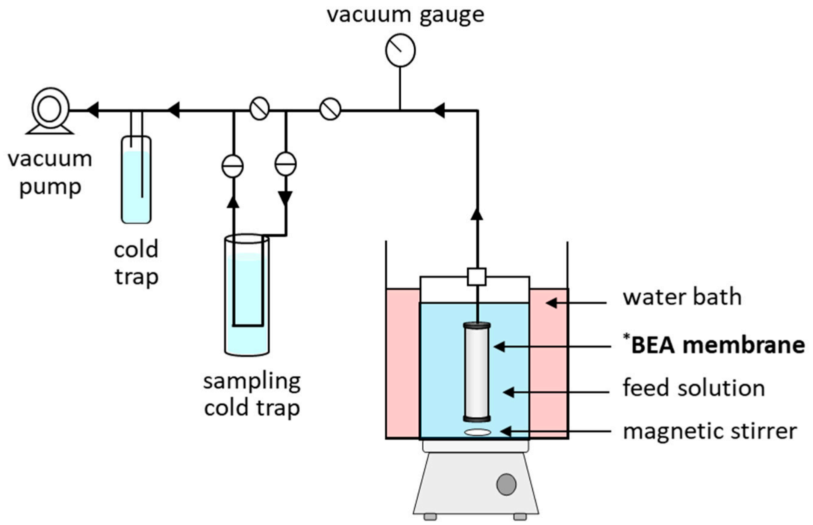

Figure S1. Experimental instrument used for measuring pervaporation performance.

Table S1. Effect of NH_4F/SiO_2 ratio of synthesis gel on the PV performance for butanol/water mixtures.

Samples	Synthesis Gel Composition *		Separation Factor	Flux [$kg \cdot m^{-2} \cdot h^{-1}$]
	y	NH_4F/SiO_2 Ratio		
M3	0.45	5.63	32.3	2.28
M5	0.50	6.25	102	0.79
M6	0.60	7.50	112	0.45
M7	0.70	8.75	229	0.62

* SiO_2:TEAOH:NH_4F:H_2O = 0.08:0.5:y:8.0.
